# RNAsnp: Efficient Detection of Local RNA Secondary Structure Changes Induced by SNPs

**DOI:** 10.1002/humu.22273

**Published:** 2013-01-11

**Authors:** Radhakrishnan Sabarinathan, Hakim Tafer, Stefan E Seemann, Ivo L Hofacker, Peter F Stadler, Jan Gorodkin

**Affiliations:** 1 Center for non-coding RNA in Technology and Health, University of CopenhagenFrederiksberg, Denmark; 2 Department of Veterinary Clinical and Animal Sciences, University of CopenhagenFrederiksberg, Denmark; 3 Bioinformatics Group, Department of Computer Science, and Interdisciplinary Center for Bioinformatics, University of LeipzigLeipzig, Germany; 4 Department of Theoretical Chemistry, University of ViennaWien, Austria; 5 Bioinformatics and Computational Biology Group, University of ViennaWien, Austria; 6 Max Planck Institute for Mathematics in the SciencesLeipzig, Germany; 7 Fraunhofer Institut für Zelltherapie und Immunologie – IZILeipzig, Germany; 8 Santa Fe InstituteSanta Fe, New Mexico

**Keywords:** RNA secondary structure, structural disruption, gene regulation, disease

## Abstract

Structural characteristics are essential for the functioning of many noncoding RNAs and *cis*-regulatory elements of mRNAs. SNPs may disrupt these structures, interfere with their molecular function, and hence cause a phenotypic effect. RNA folding algorithms can provide detailed insights into structural effects of SNPs. The global measures employed so far suffer from limited accuracy of folding programs on large RNAs and are computationally too demanding for genome-wide applications. Here, we present a strategy that focuses on the local regions of maximal structural change between mutant and wild-type. These local regions are approximated in a “screening mode” that is intended for genome-wide applications. Furthermore, localized regions are identified as those with maximal discrepancy. The mutation effects are quantified in terms of empirical *P* values. To this end, the RNAsnp software uses extensive precomputed tables of the distribution of SNP effects as function of length and GC content. RNAsnp thus achieves both a noise reduction and speed-up of several orders of magnitude over shuffling-based approaches. On a data set comprising 501 SNPs associated with human-inherited diseases, we predict 54 to have significant local structural effect in the untranslated region of mRNAs. RNAsnp is available at http://rth.dk/resources/rnasnp.

## Introduction

Distinctive structural features are a prerequisite for the proper function of many noncoding RNAs and *cis*-acting regulatory elements. Prime examples are tRNAs and rRNAs, which are among the structurally most highly conserved genes. The strong stabilizing structural selection [Piskol and Stephan, 2011] on those RNAs becomes particularly evident when comparing sequences, as a large number of the substitutions found in those RNAs are in fact compensatory mutations [Meer et al., 2010; Wu et al., 2009]. A plethora of different RNA families and classes [Gardner et al., 2011] shows the same behavior. Several computational methods utilize this effect very successfully to detect functional RNA structures by homology [Menzel et al., 2009; Nawrocki et al., 2009] and de novo from comparative genomics data [Gesell and Washietl, 2008; Gorodkin et al., 2010;Pedersen et al., 2006; Torarinsson et al., 2006; Washietl et al., 2005].

SNPs can alter the structure and thereby the function of the RNA molecules. Well-known pathogenic mutations render mitochondrial tRNA dysfunctional by disrupting their structures causing a variety of severe diseases [Wittenhagen and Kelley, 2003; Yarham et al., 2010]. Several SNPs in microRNAs (miRNAs) are known to affect processing and or targeting [Gong et al., 2012], whereas SNPs in and around miRNA binding sites and other small RNAs can induce local structural changes that affect RNAi-mediated regulatory function [Hariharan et al., 2009; Thomas et al., 2011]. Similarly, SNPs in both the coding [Bartoszewski et al., 2010; Duan et al., 2003; Nackley et al., 2006; Shen et al., 1999] and noncoding [Chatterjee and Pal, 2009; Meplan et al., 2008; Naslavsky et al., 2010] regions of mRNAs have been shown to affect the mRNA secondary structure in a way that causes aberrant gene regulation, see also Chen et al. (2006) for an extensive review. Such SNPs can frequently alter the global structure of the untranslated (UTR) [Halvorsen et al., 2010; Martin et al., 2012]. Mutations that impair replication [You et al., 2004], translation initiation [Tang et al., 1999], and splicing [Abbink and Berkhout, 2008] have also been described in RNA viruses that typically have a very high mutation tolerance. A meta-analysis of SNPs associated with human disorders shows that many of them are located in noncoding positions harboring small RNAs [Glinskii et al., 2009], see also Mattick (2009).

The effect of (point-)mutations on RNA secondary structure was studied in much detail already two decades ago, revealing two—seemingly conflicting—principles: (1) the existence of extensive neutral networks along which RNA sequences can evolve without changing the structures, and (2) a high degree of fragility so that a fraction of the possible mutations leads to very large structural changes [Fontana et al., 1993; Schuster et al., 1994]. With the availability of large amounts of variation data there has been an increasing interest in computational tools to quantify the effects of SNPs in more detail. Several tools use graph-theoretical descriptors to evaluate the effect, for example, RNAmute [Barash, 2003; Churkin and Barash, 2006] and RDMAS [Shu et al., 2006]. SNPfold [Halvorsen et al., 2010] uses the Pearson correlation of pairing probabilities of wild-type and mutant to quantify the structural effect of the SNP and to identify “RiboSNitches,” that is, SNPs with particularly dramatic effects on the RNA structure. The corRna web server [Lam et al., 2011] adheres to the same strategy but uses RNAmutants [Waldispühl et al., 2008] to compute the structural ensembles for the entire k-neighborhood of the reference sequence. Rchange [Kiryu and Asai, 2012], differs from the above approaches as it does not consider the base pair probabilities but compares the energy difference between the structural ensembles of the wild-type and mutant sequence. All these approaches employ global predictions of secondary structures or ensemble of secondary structures and hence require rather large computational resources. The deluge of variation data generated, for example, in the HapMap [The International HapMap Consortium, 2010], the International Cancer Sequencing Project (ICGC) [The International Cancer Genome Consortium, 2010], or the 1001 genome project [Cao et al., 2011], however, create a need for computationally more efficient solutions.

In many cases, structural effects are limited to local domain. For example, the mutations found in the Internal Ribosome Entry Sites (IRES) of the human p53 mRNA cause mostly local changes within the IRES, inhibiting the binding of *trans*-acting factors essential for translation [Grover et al., 2011]. Some disease-associated SNPs also have been reported to cause local structural changes affecting gene expression [Hill and Reynolds, 2011; Siala et al., 2010]. Such localized changes, however, are not detectable with global measures of structural difference, in particular when long RNA sequences are under consideration. Here, we investigate different ways of obtaining localized measures of structural dissimilarity and describe RNAsnp, a tool geared toward evaluating SNP effects on large data sets that is more sensitive to local structural change than the available methods.

## Materials and Methods

We introduce and compare several quantities that measure the effect of a small change in RNA sequence on the secondary structures. Rather than quantifying the explicit difference between predicted minimum free energy (MFE) structures, we consider the difference of the distribution of structures. These (dis)similarity can be applied either globally or restricted to a particular local context.

### RNA Folding

We recall that for each RNA sequence *x*, there is a well-defined set of secondary structures *Ω*(*x*) that obey the base pairing rules. For each of the secondary structures *ψ* ∈ *Ω*(*x*), a folding energy *E*(*ψ*, *x*) can be computed from the so-called Turner energy model [Mathews et al., 2004]. The structure *ψ* occurs with probability *p_x_*(*ψ*) = exp(−*E*(*ψ*,*x*)/*RT*)/*Z_x_* in the Boltzmann ensemble. The normalization constant *Z_x_* = ∑*_ψ_*_∈*Ω*(*x*)_ exp(−*E*(*ψ*,*x*)/*RT*) is the partition function of the ensemble. The ensemble of secondary structures is well described by the base pairing probability matrix *P*, whose entries


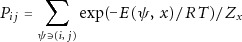
(1)

are the probabilities that the nucleotides at sequence positions *i* and *j* form a base pair in thermodynamic equilibrium. In practice, these quantities do not need to be computed by summing over all structures as in Eq. (1). Instead, the entire matrix *P* can be computed efficiently with McCaskill's algorithm [McCaskill, 1990]. To this end, we use RNAfold as well as RNAplfold [Bernhart et al., 2006], a local variant that averages the base pair probabilities over overlapping sequence windows. Both tools are components of the Vienna RNA Package [Hofacker et al., 1994; Lorenz et al., 2011].

### Structural (Dis)Similarities

The differences in the structural ensembles of a reference sequence and a mutant can be measured as a (dis)similarity of their respective base pairing probability matrices *P* = (*P_ij_*) and 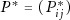. We focus here on SNPs, so that the correspondence of sequences positions between the two sequences is obvious a priori. We remark that this is not necessarily the case for insertions, deletions, or other types of variation that change sequence length and hence require the computation of an alignment to compare the structural ensembles [Hofacker et al., 2004]. A wide variety of (dis)similarity measures has been discussed in the literature. Probably the most simple one is the Euclidean base pairing distance *d*(.,.) defined by


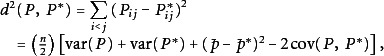
(2)

where 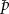 and 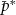 are the average base pairing probabilities of reference and mutant, respectively, and cov(*P*,P*) is the covariance of the distributions *P* and P*. It is closely related to the Pearson correlation coefficient


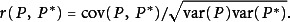
(3)

The normalization of *r* removes the dependence of the overall strength of the pairing and thus tends to overemphasize the effect of structural changes in flexible RNAs without well-defined secondary structures.

Instead of comparing the base pairing matrices directly, Halvorsen et al. (2010) consider the vectors *π* and π* of position-wise pairing probabilities *π_i_* = ∑*_j_P_ij_*. A Euclidean distance *d*^2^(*π*,π*) and a correlation coefficient *r*(*π*,π*) is defined as in Eq. (3). Alternatively, one might want to distinguish the probabilities that a base is paired upstream (*ξ*^<^), downstream (*ξ*^>^), or remains unpaired (*ξ*^0^)


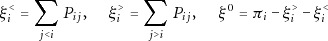
(4)

Distance measures derived from these three quantities have been used in particular in specialized structure-aware sequence alignment algorithms for structured RNAs [Bonhoeffer et al., 1993; Dalli et al., 2006; Kruspe and Stadler, 2007].

An appealing alternative is to compute the Euclidean distance *δ* between the two distributions of structures directly, thus avoiding the detour via base pairing probability matrices. Although the definition of *δ* calls for a sum over the exponential number of possible secondary structures, it can be computed in polynomial time:


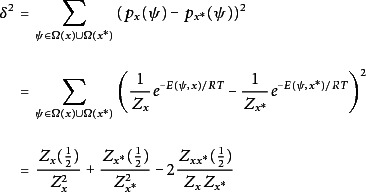
(5)

The symbols 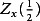 and 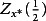 denote the partition functions for wild-type and mutant computed with a value of the Boltzmann constant artificially reduced by a factor 1/2. The last term can be rewritten as


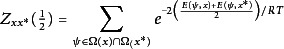
(6)

This is the modified partition function over the alignment of *x* and x* admitting only those base pairs that can be formed by both structures. The energy of each structure *ψ* is computed as the average over the two sequences. This is the same as averaging over the energy contributions for the structural elements of the two input structures, that is, the energy model used in alignment folding algorithms such as RNAalifold [Hofacker et al., 2002]. In fact, the partition function variant of RNAalifold computes 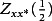 when both the bonus term for sequence covariation and the tolerance for nonstandard base pairs are set to 0. A local version can be made if we only consider partition functions on respective subsequences, but is beyond the scope of this work.

### Local Structural (Dis)Similarities

It is well known that the accuracy of RNA secondary structure predictions is far from perfect. In particular long-range base pairs have limited accuracy [Churkin and Barash, 2006]. In a cell, furthermore, most RNA molecules, in particular messenger RNAs, interact with a wide variety of proteins, further limiting the predictability of long-range effects. It appears reasonable, therefore, to concentrate on the local RNA structure in the neighborhood of the SNP. Fixing a sequence interval [*k*,*l*], it is straightforward to modify the definitions above to restricted quantities such as


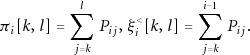
(7)

Similarly, we can define a 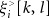. The corresponding distances *d*_[*k*,*l*]_(.,.) and correlation coefficients *r*_[*k*,*l*]_(.,.) are then computed by summing only over sequence positions in the interval [*k*,*l*].

It is less trivial, however, to determine which sequence interval [*k*,*l*] should be selected. Conceptually, it appears to be most useful to focus on the interval that exhibits the largest structural impact of the SNP, for example,


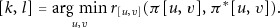
(8)

The sequence interval that maximized the effect for one of the other (dis)similarity measures introduced above is determined analogously.

At face value, this minimization is rather expensive because for each index pair *u*,*v*, the values of *π_i_*[*u*,*v*] need to be determined. The naïve evaluation of Eq. (7) can be replaced by a recursive scheme (Supp. Fig. S1). Consider sequence positions *k* < *i* < *l* and denote by *M_ik_* and *N_il_*, the probabilities that *i* has a pairing partner in the interval [*k*,*i* – 1] and the interval [*i* + 1,*l*], respectively. Clearly, these auxiliary variables satisfy *M_ik_* = *M_i_*_(*k* − 1)_ + *P_ki_* and *N_il_* = *N_i_*_(*l* + 1)_ + *P_il_*. Obviously, for all *k* < *i* < l, we have *π_i_*[*k*,*l*] = *M_ik_* + *N_il_*, 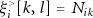, and 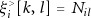. Precomputing *M* and *N* thus allows the evaluation of distances and correlation coefficients in linear time for each sequence interval.

It appears natural to apply one or more filters to reduce the noise in the data. The simplest one is to ignore all base pairs that are less likely than a user-defined threshold *P_ij_* < *p*_thr_.

Local regions of interest, furthermore, should be reasonably self-contained, that is, the base pairs should predominantly connect nucleotides that are located inside of the region of interest. We therefore restrict the optimization to sequence intervals [*k*,*l*] with the property that base pairs rarely cross the boundaries of the interval. A similar approach is taken by Dotú et al. (2010) to identify locally folded regions or modules. To quantify the self-containedness of a region, we compare the expected number


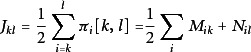
(9)

of base pairs inside [*k*,*l*] with the expected number of nucleotide within [*k*,*l*] that are paired with nucleotides outside of [*k*,*l*]


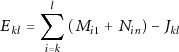
(10)

where 1 and *n* are the start and end of the sequence. An interval [*k*,*l*] is included only if the pairing within outweighs the pairing across the boundary, that is, if


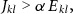
(11)

holds for either the reference or the mutant structure. Extensive testing (Supp. Fig. S2) showed that *α* = 1 is a good default value for the threshold parameter.

A considerable amount of computational resources can be saved by replacing the exact optimization over all intervals [*u*,*v*] by a simpler procedure when large sequences are considered for comparison. To this end, we furthermore modify the distance measure to consider only base pairs with limited span. For instance, we set


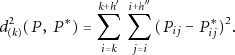
(12)

This expression contains two user defined parameters. The first one, *h*′, is the length of the local structural element that we expect to have an impact on the function. The effect of the mutation is integrated over the short interval [*k*,*k* + *h*′] only. We use a small value *h*′ = 20, motivated by the size of miRNA binding sites or protein binding motifs [Bahadur et al., 2008]. The second parameter, *h*″, is the length of the interval over which the local structural changes are evaluated, that is, the maximal span of a base pair. Guided by our previous work on computing accessibilities for siRNA design [Tafer et al., unpublished ], we use *h*″ = 120. A similar window length was reported by Lange et al. (2012), who showed that most of the base pairs in the structured RNAs (in particular *cis*-regulatory elements in mRNAs) have base pair span not larger than 100 nts. We note that 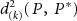 can also be computed recursively:


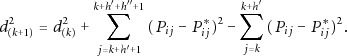
(13)

The restriction of the local base pairs with a maximal span *h*″ allows us to use RNAplfold [Bernhart et al., 2006], a scanning version of the partition function folding algorithm that is geared toward genome or transcript-wide applications. The use of RNAplfold also provides an additional benefit: this algorithm computes average base pairing probabilities over all sequence windows of limited size that contain the base pairs. Hence, it emphasizes, for each individual sequence, those structures that are consistently predicted and that are more likely to be functional.

We first determine the position k* that maximizes 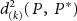. Then, we compute the maximal dissimilarity starting at k*, that is,


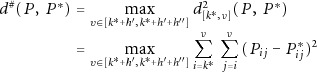
(14)

As shown in Supp. Figure S3, this drastically reduced the number of intervals that need to be considered.

Before proceeding, let us summarize the plethora of different measures that are based on the base pairing probability matrix *P* introduced above. These can be classified in several ways: (1) in addition to *P* itself, we consider several marginal distributions such as the probabilities *π* of individual bases being paired. (2) These distributions are compared either by a Pearson correlation coefficient *r*(.,.) or a Euclidean distance *d*(.,.). (3) Each of these measure can be computed either globally (*r* or *d*), locally on the optimal interval (*r*_min_ or *d*_max_), or approximated with restricted base pair spans in the “scanning version” (*r^#^* or *d^#^*). The symbols are summarized in Table 1.

**Table 1:** Summary of the Notation for (Dis)Similarity Measures Between Base Pair Probabilities of Wild-Type and Mutant

Probability distribution	
*P*	Full base pairing probabilities
*π*	Position-wise pairing probabilities
*ξ*^<^	Position-wise, upstream
*ξ*^>^	Position-wise, downstream
*ξ*^<>^	Position wise, distinguishing up- and downstream
Similarity measure	
*r*	Pearson correlation
*d*	Euclidean distance (*L*^2^ norm)
Optimization	
*r*, *d*	Global
*r*_min_, *d*_max_	Best local interval
*r*^#^, *d^#^*	Approximate optimization for scanning

### Comparison of Local (Dis)Similarity Measures

**Figure 1:**
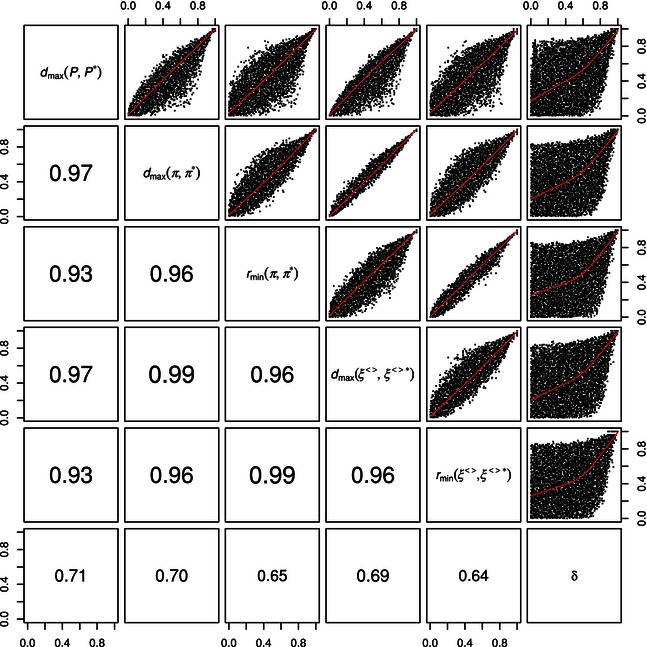
The scatter plot shows the rank correlation between various (dis)similarity measures, which were tested on 7,000 random sequences of length 400 nts with G+C contents between 20% and 80% and a SNP introduced at position 200. The local measures *d*_max_ and *r*_min_ based on the base pairing probability matrix *P* and its marginal distributions *π* and *ξ*^<>^ (see Table 1) correlate well with each other. The distance *δ* of the Boltzmann distribution, on the other hand, behaves quite differently from the above *P*-derived measures.

To determine to what extent these (dis)similarity measures are correlated, we used a set of 7,000 sequences of length 400 that are generated randomly with different G+C contents ranging from 20% to 80% in steps of 10%. For each sequence, we computed the base pairing probability matrices *P* using RNAfold, for all three possible substitutions at position 200. From *P*, the marginal distributions *π* and *ξ*^<>^ (see Table 1) were computed. The difference between the wild-type and mutant was calculated using the different local (dis)similarity measures *d*_max_ and *r*_min_ based on *P* and its marginal distributions *π* and *ξ*^<>^. The results obtained for each measure were ranked. The different local (dis)similarity measures based on base-pairing probabilities, with the exception of *r*_min_(*P*,P*), correlate with each other (see Fig. 1). The corresponding global version including *r*(*P*,P*) also correlate with each other (see Supp. Fig. S4).

In addition, we investigated the Euclidean distance *δ*, Eq. (5), between the distributions of wild-type and mutant structures. Surprisingly, it correlates poorly with the distances of the distributions derived from *P* (last column in Fig. 1). A possible explanation is that the measures based on *P* identifies structural differences at the nucleotide level, whereas the Euclidean distance *δ* identifies differences in the distribution of structural ensembles between wild-type and mutant.

### Empirical *P* Values

A distance measure *d* between *P* and P* provides information on the significance of a structural change only when compared with some background distribution. In principle, this background distribution can be generated by shuffling the input sequences and computing the SNP effects for every shuffled copy. Because the RNA folding algorithms have cubic runtime complexity, this is a computationally very demanding task.

To reduce the computational runtime of RNAsnp, extensive background-distribution tables for a variety of score are provided together with RNAsnp. The background distributions were computed based on a set of 7,000 random sequences with lengths ranging from 400 to 1,600 nts in steps of 100 nts and G+C content varying between 20% and 80% in steps of 10% (see Gruber et al., 2010 for more details). We generated all possible substitutions at each nucleotide position and computed the distance measures summarized in Table 2. About 156 CPU (core) years of computation time was required to compile the necessary simulation data. However, with the help of massive parallel processing server (HP Bl280cG6) with 2,000 processors, the entire computation was accomplished in 1 month.

**Table 2:** Summary of Structure Distance Measures Implemented in RNAsnp

Measure	Folding	Distr.	*ρ*
*d*_max_	RNAfold	Gumbel	0.9982
*d*_max_	RNAplfold	Gumbel	0.9999
*d*^#^	RNAplfold	Gumbel	0.9999
*r*_min_	RNAfold	Beta	0.9986

The first column represents the distance measure, the second column lists the folding program used to computing the base pair probabilities. The remaining columns give the type of distribution used to fit the empirical *P*-value distribution and the correlation coefficient *ρ* between the fitted *P* values and the rank-based *P* values computed for a set of random sequences. The fitted *P* values are computed using the parameters from fitted distribution.

**Figure 2:**
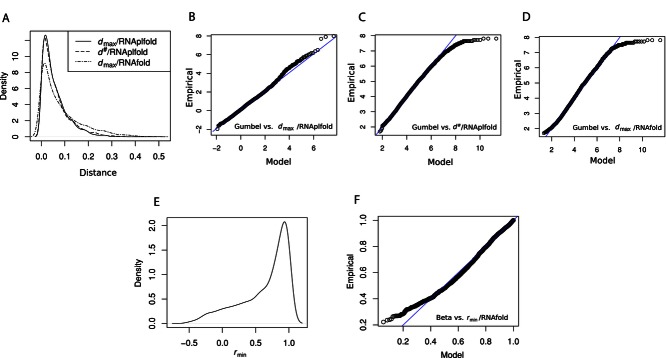
(**A**) Density plot showing the distribution of three distance values calculated using random sequences of length 400 nts, a G+C content between 50% and 60% and the SNP position at 200 position. (**B**) Quantile–quantile plots of the transformed *d*_max_ computed from the base pair probabilities returned by RNAplfold against the fitted gumbel distribution. (**C**) Same as (**B**) for *d*^#^. (**D**) Same as (**B**) but the *d*_max_ computed with the base pair probabilities returned by RNAfold. (**E**) The density plot showing the distribution of *r*_min_ computed from the base pair probabilities returned by RNAfold and (**F**) quantile–quantile plot showing the transformed *r*_min_ against the beta distribution.

For a given distance measure, length, G+C content, and SNP position within the sequence, the dissimilarity scores *d* closely follow an extreme value distribution. As an example, Figure 2 shows the distribution of distance values that are calculated using random sequences of length 400, a G+C content between 50% and 60%, and a SNP at position 200. Consequently, 1 – log(*d*) approximately follows a Gumbel distribution. The gum.fit function of the ismev [Coles, 2001] package for the R statistics program [R Development Core Team, 2012] was used to compute maximum-likelihood estimates of the parameters *μ* and *σ* for every sequence length, G+C content, SNP position. Although the fitted distribution does not match perfectly the empirical *P* values for RNAfold/*d*_max_ according to the Kolmogorov–Smirnov test, the deviations only concern structures with very high discrepancies where empirical *P* values cannot be estimated accurately. The comparison of fitted *P* value (described below) and rank-based *P* value shows a very high correlation (Supp. Fig. S5). The *P* value of an observed dissimilarity value *d* can now be computed efficiently as


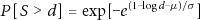
(15)

using the tabulated values of *μ* and *σ*.

The distribution of the correlation coefficients *r*, Figure 2E, closely follows a beta distribution after the transformation *y* = (*r* + 1)/2. The transformed can then be fitted to a beta distribution (Fig. 2F) using the R function fitdistr from MASS package [Venables and Ripley, 2002]. The comparison of the fitted *P* value (computed using the parameters of the fitted beta distribution) and the rank *P* value shows high correlation (see Supp. Fig. S5).

### Implementation of RNAsnp

**Figure 3:**
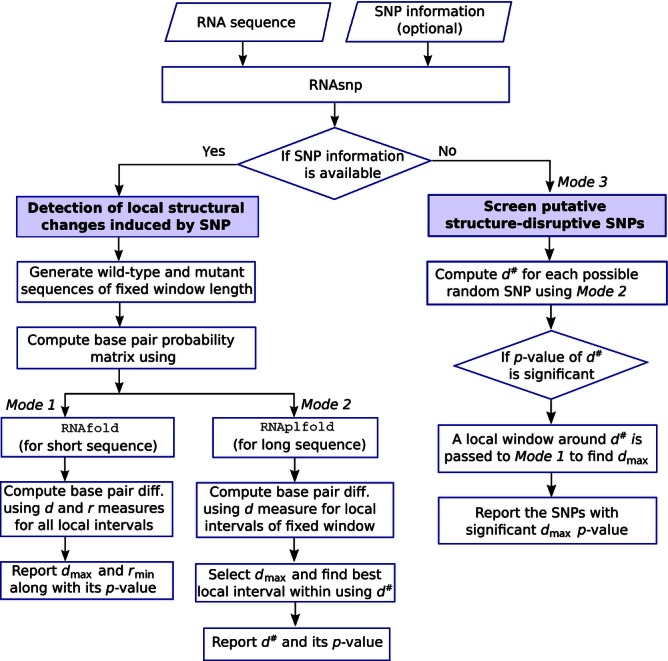
Flowchart of RNAsnp program. The options “Mode 1” and “Mode 2” help to detect the SNP induced local structural changes in a given RNA sequence. The choice of the modes depends on the length of the input sequence. The option “Mode 3” helps to screen the putative structure-disruptive SNPs in an RNA sequence. This is achieved by testing the effect of all three possible substitutions at each nucleotide position. This mode facilitates an effective screening of putative structure-disruptive SNPs from transcripts or genome sequences.

The program RNAsnp offers three different modes (Fig. 3). The first mode is designed to compute the effect of SNPs by using global folding. This option should be used only for short input sequences as the base pairing probabilities are calculated here using the global folding method RNAfold. Further, the structural difference between the wild-type and the SNP allele is assessed by computing the correlation coefficient [using Eq. (3) and Eq. (7)] and the Euclidean distance [using Eq. (2)] for all sequence intervals with a minimum length of 50 that have self-contained base pairs. The minimum length was chosen after a careful analysis with different length cutoffs (Supp. Fig. S6). Finally, the interval with maximum base pairing distance or minimum correlation coefficient and the corresponding *P* value are reported.

The second mode is designed to compute the effect of SNPs on large sequences. To this end, we first compute the local base pair probability using RNAplfold with the parameters -*W* 200 and -*L* 120. In a second step, the base pairing distance [using Eq. (12)] is computed for all sequence intervals of fixed length *h*′ and *h*″. The sequence interval with maximum base pair distance (*d*_max_) is considered for reestimation using the exact optimization within the local window according to Eq. (14). The significance of the dissimilarity measure is computed from the tabulated values of *μ* and *σ*. A similar procedure for the correlation coefficients has not been made available because they perform poorly on the available test data, see Supp. Figure S7.

The third mode (or screening mode) is the combination of the above two. It is intended to determine the positions of putative structure-disruptive SNPs in a transcript or genomic sequence using the brute-force search. First the *d^#^* distance is computed based on the base pairing probabilities obtained from RNAplfold for all possible substitutions at every nucleotide position. The corresponding *P* values are computed from the tabulated values of *μ* and *σ*. For candidate SNPs with sufficiently small *P* values, a local window is extracted and passed to RNAfold to compute the global base pairing probabilities of the subsequence, which is then rescored using the more accurate *d*_max_ measure as in the first operating mode. We further developed a PERL script that analyses the output of the screening mode and plots the regions of highly occurrent low *P*-value SNPs. Further, it also returns how many times a region was reported to be structurally disrupted.

The RNAsnp software requires an RNA (or DNA) sequence as input. Optionally, the SNP(s) to be analyzed and the mode of operation can be specified. By default the program uses a window of 400 nts, ±200 nts around the SNP position, to compute the base-pairing probability. This default value -winsizeFold = 200 can be changed between 200 and 800 (inclusive) in multiples of 50. This restriction is necessary to keep the size of parameter tables for the *P* values calculations manageable.

### Datasets

We use three data sets to assess the difference between global versus local structure disruption, to evaluate different measures and to search for disruptive SNPs in human UTR.

#### *SNP*s with reported structural effects

To evaluate the performance of RNAsnp, a set of SNPs with known impact on the RNA structure is required. Unfortunately such data are still very scarce. We compiled a list 30 known SNPs (Supp. Table S1) for which an effect on the RNA structures has been discussed. Of these 30 SNPs, 25 SNPs are from human mRNAs, one is from a rat mRNA, and the remaining four are from viral sequences. The structural changes were verified experimentally in only four of them using chemical or enzymatic structure probing methods [Grover et al., 2011; Shen et al., 1999; Westerhout et al., 2005; You et al., 2004]. In the remaining cases, only a careful computational analysis has been reported.

#### *SNP*s associated with human heritable diseases

A comprehensive dataset of 514 disease-associated SNPs was extracted from Halvorsen et al. (2010). These SNPs are a subset of the Human Gene Mutation Database (HGMD), which contains the collection of mutations and polymorphisms associated with human-inherited diseases [Stenson et al., 2009]. These 514 SNPs were mapped to 292 Refseq (mRNA and noncoding RNA) sequences downloaded from the UCSC genome browser site (http://genome.ucsc.edu) [Karolchik et al. 2008]. In mRNAs, the SNPs are located either in 5′ or 3′ UTRs. Of these 514 SNPs, 13 were removed as they are already included in the collection of SNPs with reported structural effects in the previous paragraph.

#### Known *UTR*s *SNP*s in human m*RNA*s

For each of the 21,081 human protein coding genes available in Ensembl 66 [Kinsella et al., 2011], the longest transcript was selected. A total of 263,248 UTR SNPs from the NCBI dbSNP Build 132 were mapped to these transcripts variants, resulting in 195,916 SNPs mapping to 15,257 genes. Because more than two alleles are reported for some SNPs and some SNPs map to more than one transcript, this amounts 201,213 distinct mutants, of which 27,687 were located in the 5′ UTR and 173,526 were located in the 3′ UTR. From the 1,686 disease-associated UTR variants collected in GWASdb [Li et al., 2012], 1,422 SNPs were mapped to our set of transcripts, 152 and 1,270 being located in the 5′ and 3′ UTR, respectively.

For each data set, the SNPs were formatted to HGVS nomenclature. Thus, for mRNA sequences, the nucleotide numbering reflects cDNA numbering with +1 corresponding to the A of the ATG translation initiation codon in the reference sequence. In addition, the nucleotide numbering of 5′ and 3′ UTR regions were preceded with “-” and “*” symbols, respectively. For noncoding RNAs, the nucleotide positions numbered relative to transcription start site.

## Results

### Prediction of Structural Disruption

**Figure 4:**
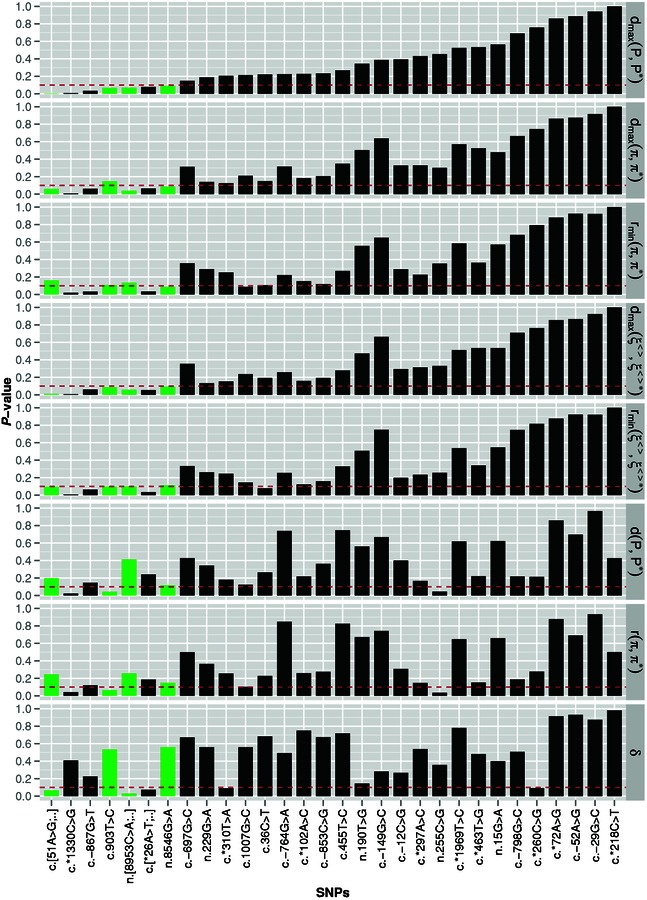
Significance of structural effects as predicted by the local (dis)similarity measures (*d*_max_ and *r*_min_ using different probability probabilities: *P*, *π*, and ξ^<>^) and distance (*δ*) between the distribution of structures for the 30 SNPs with reported secondary structure changes. See Table 1 for more details about the symbols. The *P* values are shown as bars and the dashed line represents the selected threshold value 0.1. The four experimentally validated examples are indicated in green/gray. The SNPs were described according to HGVS nomenclature.

All distance and correlation measures were evaluated in terms of *P* values assigned to the 30 SNPs with reported structural effect. Figure 4 shows that *d*_max_ and *r*_min_ assign similar *P* values to each SNP, whereas the significance values returned by the Euclidean distance *δ* between the distributions of structures show no such correlation. This might be explained by the fact that these significant local structural changes share large parts of the structure distribution between wild-type and mutant. Conversely, *δ* does not depend on the base pair distances between the dominating structure in the two ensembles but only on whether the dominating structures are identical or distinct.

**Figure 5:**
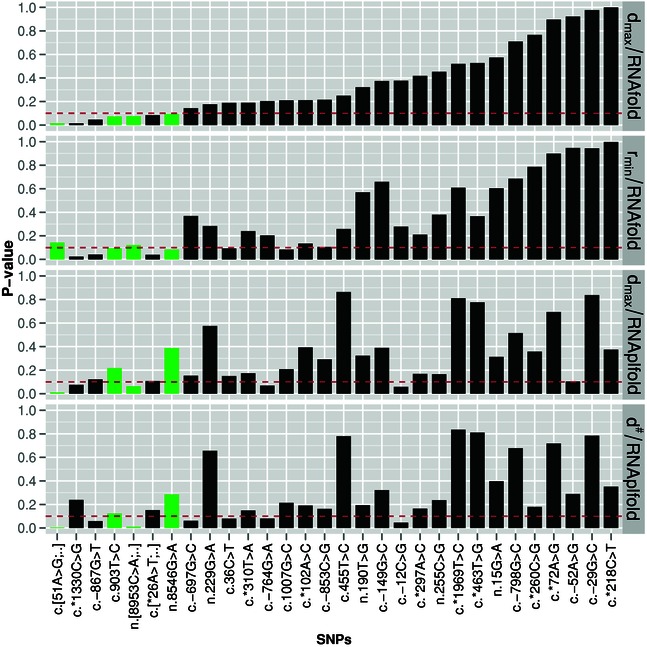
Significance of structural effects using RNAsnp (Modes 1 and 2) for the data set of 30 SNPs with reported secondary structure changes. The *P* values are shown as bars and the dashed line represents the selected threshold value 0.1. The four experimentally validated examples are indicated in green/gray. The SNPs were described according to HGVS nomenclature.

Despite the substantial correlation between the different dissimilarity measures based on the base pairing probabilities there appears to be a difference in the sensitivity. Using a *P*-value threshold of *P* ≤ 0.1, the localized distance *d*_max_ computed from the global base pairing matrix, that is, with RNAfold, recognized all four experimentally validated SNPs, whereas the most reliable alternatives identified only two of them, see Table 3. On the other hand, all measures identify essentially the same local region of structural change and match to the local region reported in literature. The *P* values of the local measures, furthermore, are smaller than the *P* values of the global measures *d*, *r*, and SNPfold, see Table 3. Note that the *P* value of the global measures *d* and *r* are still smaller than those of SNPfold. The higher *P* values for SNPfold may in part be explained by the different approaches used to generate the background distribution in RNAsnp and SNPfold for the *P*-value calculation. Figure 5 shows that on the remaining 26 SNPs with only putative structural effects another three SNPs reach *P* < 0.1 when *d*_max_ is used with either global or local folding, whereas five candidates pass the threshold for *d^#^* with local folding and *r*_min_ with global folding. Consistent predictions are obtained only for the SNPs c.*1330C>G, c.-867G>T, and c.-764G>A. An additional indication for structural disruption of these SNPs is the report by Teresi et al. (2007) that c.-867G>T and c.-764G>A are associated with the Cowden Syndrome.

**Table 3:** The Results of RNAsnp and SNPfold for the Four Cases with Experimentally Verified Structures

				*d*_max_/RNAplfold		*d*^#^/RNAplfold		*d*_max_/RNAfold		*r*_min_/RNAfold		*D*/RNAfold	*r*/RNAfold	SNPfold
										
References^a^	Gene^b^	SNP	Local reg. c	max*_k_*	*P* value	Interval	*P* value	Interval	*P* value	Interval	*P* value	*P* value	*P* value	*P* value
1	p53	c.[51A>G;54A>C;57T>C]	257–262	241	0.009	241–285	0.004	241–290	0.015	231–280	0.142	0.197	0.245	0.664
2	NS5B	n.[8953C>A;8955T>G]	9,271–9,294	9,270	0.062	9270–9,298	0.009	9,261–9,310	0.074	9,268–9,317	0.121	0.412	0.257	0.359
3	AARS	c.903T>C	980–1,032	994	0.217	998–1,052	0.123	975–1,025	0.072	998–1,052	0.093	0.042	0.065	0.077
4	Nef	n.8546G>A	8,512–8,579	8,517	0.387	8,517–8,555	0.285	8,518–8,576	0.094	8,518–8,567	0.083	0.116	0.148	0.293

The SNPs were described according to HGVS nomenclature.

a (1) Alteration of RNA replication in HCV [You et al., 2004], (2) tumor formation [Grover et al., 2011], (3) HIV-1 resistance against RNAi [Westerhout et al., 2005], and (4) alteration of alanyl-tRNA synthetase expression in human [Shen et al., 1999].

b Refseq/Accession ids: NS5B – AJ238799.1, p53 – NM_001126114.2, Nef – K02013.1, AARS – D32050.1.

c Position of the local regions reported in literature. The start and end values are represented with respect to the sequence position of full length mRNA sequence.

Furthermore, the MFE and structural-ensemble based predictions of SNP effects were studied with the same data set of 30 SNPs with reported structural effect (Supp. Fig. S8). It shows that the *P* values from the local measures based on the structural ensemble are in general smaller than the *P* values derived from the MFE structure. The comparison of two *P*-value distributions using Wilcoxon rank sum test, however, shows no significant difference (*P* > 0.2), probably due to the small number of available data. In addition, the measures of RNAsnp based on based pairing probability of structural ensemble were compared with the measures of Rchange [Kiryu and Asai, 2012], using the four known SNPs with experimentally validated structural effect. Because Rchange can handle either single or double mutations, only three out of four SNPs could be studied. The empirical *P* values were calculated for the results of Rchange to compare with RNAsnp *P* values (see Supp. Table S2). At a significance level of 0.1, Rchange predicted one out of three SNPs to have significant structural effect, whereas the measures of RNAsnp, *d*_max_/RNAfold predicted all three cases and *r*_min_/RNAfold predicted two cases (see Supp. Table S2).

### Structural Effects of Disease-Associated SNPs

The structural effect of disease-associated SNPs was studied by looking at the 501 disease-associated SNPs compiled by Halvorsen et al. (2010). Using a significance threshold of 0.1, 34 disruptive SNPs were found with *d*_max_/RNAfold and 40 SNPs with *r*_min_/RNAfold, see Supp. Table S3. The 20 candidates listed in Table 4 are shared between the lists. Only three of these 20 SNPs are predicted by SNPfold [Halvorsen et al., 2010] to have a significant global structural effect.

**Table 4:** List of Disease-Associated SNPs that are Predicted to have Significant Local Structural Effect (*P* < 0.1) Based on RNAfold with the Scores *d*_max_ and *r*_min_

						*P* value	
						
Disease/phenotype	Gene	HGMD Accession	GenBank Accession	NTs	SNP	*P*(*d*_max_)	*P*(*r*_min_)
Pseudohypoaldosteronism	NR3C2	CR030126	NM_000901.4	5,898	c.-2C>G	0.017	0.022
Hypertension	EDN2	CR994679	NM_001956.3	1,243	c.^*^390G>A	0.036	0.021
Obesity	CNR1	CR073542	NM_033181.3	5,373	c.^*^2394A>G	0.032	0.036
Myocardial infarction	GP1BA	CR022116	NM_000173.5	2,463	c.-5T>C	0.040	0.037
Colorectal cancer	INSR	CR082021	NM_001079817.1	9,023	c.^*^104A>G	0.042	0.030
Graves’ disease	FCRL3	CR067134	NM_052939.3	3,019	c.-11G>C	0.011	0.042
Increased triglyceride levels	ABCA1	CR025352	NM_005502.3	10,502	c.-279C>G	0.044	0.022
Insulin resistance and hypertension	RETN	CR032443	NM_020415.3	478	c.^*^62G>A	0.045	0.043
Cartilage-hair hypoplasia	RMRP	CR063417	NR_003051.3	268	n.215A>G	0.048	0.027
Hypercholesterolemia	LDLR	CR971948	NM_000527.4	5,283	c.-14C>A	0.025	0.048
Glaucoma	CYP1B1	CR032431	NM_000104.3	5,153	c.-286C>T	0.063	0.036
Reduced transcriptional activity	NR3C1	CR016150	NM_001024094.1	6,787	c.-219C>A	0.044	0.063
HDL cholesterol levels	LIPG	CR032437	NM_006033.2	4,141	c.^*^482A>G	0.051	0.065
Factor VII deficiency	F7	CR090334	NM_019616.2	3,059	c.-44T>C	0.066	0.042
Hemophilia A	F8	CR070421	NM_000132.3	9,035	c.-112G>A	0.074	0.010
Cartilage-hair hypoplasia	RMRP	CR064472	NR_003051.3	268	n.10T>C	0.076	0.024
Von Hippel–Lindau syndrome	VHL	CR011856	NM_000551.3	4560	c.^*^7C>G	0.076	0.065
Obesity	SLC6A14	CR035766	NM_007231.3	4,564	c.^*^178C>G	0.078	0.062
Spastic paraplegia 31	REEP1	CR082030	NM_022912.2	3,853	c.^*^14C>T	0.033	0.081
Hyperferritinemia-cataract syndrome	FTL	CR061334	NM_000146.3	871	c.-178T>G	0.052	0.097

The SNPs were described according to HGVS nomenclature. SNPs, which have been predicted to have significant global structural effect using SNPfold [Halvorsen et al., 2010], are highlighted with gray background color.

To check whether the disease-associated SNPs are more disruptive than the neutral SNPs, 501 HapMap SNPs with minor allele frequency (MAF) greater than or equal to 40% and 501 dbSNP SNPs with MAF less than 1% were tested with RNAsnp (first mode). No significant difference could be found between the distribution of RNAsnp *P* value of both groups and disease-associated SNPs (see Supp. Fig. S9).

To assess the proportion of disruptive SNPs in the UTRs, RNAsnp was applied to a set of about 201,213 known SNPs in human UTRs sequences. As a pragmatic rule, we required a SNP to satisfy *P*(*d*_max_) < 0.4 in the initial assessment based on the RNAplfold predictions and *P*(*d*_max_) < 0.1 in the recomputation with RNAfold.

This was compared with the proportion of disruptive SNPs for all possible variants in the UTRs of our transcript (105,933,543 variants). A total of 1,616,550 SNPs out of the 18,374,109 5′-UTR SNPs (8.3%) and 8,207,993 out of the 87,559,434 3′-UTR SNPs (9.3%) were reported by RNAsnp (third mode) to be disruptive, reasonably close to the expected 10% at our chosen level of significance. We further looked at the distance distribution between predicted disruptive SNPs. The average distance between disruptive SNPs is close to 10 nts, whereas the median distance between two disruptive SNPs is 1 nt, indicating that predicted disruptive SNPs tend to be clustered. Among the reported known SNPs in dbSNP, structural disruption is significantly reduced (*P* < 0.005) with 2088 out of the 27,687 5′ UTR SNPs and 13,107 out of the 173,526 3′ UTR SNPs, that is, 7.5% in both samples. This is expected as structurally disrupting SNPs are probably under selective pressure and hence should be observed less frequently than neutral variants. This reduction in the number of prediction thus demonstrates that RNAsnp might pick up a real biological signal. Interestingly, the same ratio (109 of 1,422, 7.6%) is found for the known disease-associated SNPs from GWASdb [Li et al., 2012]. The details of these 109 disease-associated SNPs are listed in Supp. Table S4.

## Discussion

Genomic variation has long been known to cause a variety of diseases Manolio (2010), probably the most profound are known from nonsynonymous SNPs in protein coding sequence and transcription factor binding sites. In this study, the effects of SNPs on the local structures of non-coding and regulatory RNAs was addressed.

The impact of sequence variation on the Boltzmann ensemble of secondary structures was compared using both global and local folding approaches. This analysis showed that local measures of structural variation based directly on a comparison of base pairing probabilities are most informative. Euclidean distances of base pairing patterns, furthermore, perform better than the correlation coefficients used in earlier work as a measure of structural variation. The latter effect is probably due to the invariance of the correlation measures to the absolute strength of base pairing.

The beneficial effect of localizing the measures of structure discrepancy are explained by the fact that small variations in the pairing probabilities far away from the site of the SNP are added up and hence form a major contribution to any global discrepancy measure in particular for long sequences. We expect that these distant variations will in general play little role for the molecule's function compared with structural changes relatively close to the SNP. The restriction of the distance measurement to the local region with the largest structural change therefore serves as a means of reducing the signal-to-noise ratio. We emphasize that this does not imply that the base pairing of the SNP position itself is part of a structural rearrangement. For instance, a change in the stability of a loop in which the SNP resides can force a major refolding. Necessarily, however, the base pairs that delimit the loops to which the SNP position contributes must be strongly affected. Hence, large structural changes also have a large local component.

On the basis of these observations, we developed RNAsnp as a tool to assess the effect of SNPs on RNA secondary structures in large data sets. RNAsnp makes use of the efficient scanning variants of RNA folding algorithms provided by the Vienna RNA Package and uses precomputed tables describing the score distributions as a function of sequence length, G+C content, and SNP position, and distance or correlation measure. Thus, *P* values can be computed efficiently without the need to sample empirical score distribution from shuffled sequences. As a consequence, RNAsnp is thus fast enough to screen large data sets and even to precompute maps of SNP effects at transcriptome or even genome-wide scales, a feature not found in other tools. Compared with SNPfold, RNAsnp is able to predict the extent and exact location of the structural changes.

A general problem for the development and evaluation of tools addressing the impact of SNPs on RNA secondary structure is the limited amount of available cases of SNPs being experimentally shown to actually disrupt an RNA structure. In spite of the deep literature survey, what remained was still a limited amount of example (4) with direct experimental evidence, but an extension to some 30 examples could be made.

In Ritz et al. (2012), the capability of RNA folding algorithms to predict the relative magnitude of structural changes was compared with SHAPE reactivity data using different artificial mutations in five highly structured RNAs. This analysis showed that the predictive power of thermodynamics-based folding approaches is rather poor. This is not surprising in this case, as the dataset used contained Riboswitches known to have tertiary RNA structures that are not predicted by the common RNA folding programs. Like RNAsnp, SNPfold is based on thermodynamics structure prediction, and therefore performs similarly poorly (data not shown). However, the setting of this benchmark study is quite different from ours. We are primarily interested in detecting mutations that have large effects in long mRNA sequences, as opposed to determining the ranks of alternative, often close-by mutations.

RNAsnp assigned low *P* values to all four experimentally validated cases. This is reassuring despite the small size of the test set. We emphasize that RNAsnp does not include any machine learning or other trained component, and hence does not use a training set. It is entirely based on the “ab initio” thermodynamic model of RNA folding. Interestingly, RNAsnp does not predict a strong structural effect for many of the cases for which structural disruption has been proposed in the literature but not validated experimentally. In most of these studies only the minimum energy structure was evaluated. This leads to an overestimation of the structural effects since the MFE structures are much more sensitive to small changes in the sequence than the Boltzmann ensemble. It is well known that two distinct structures with similar energy can easily exchange their rank order with respect to energy without substantially changing their relative frequency in the ensemble.

At a significance level of *P* < 0.1, we find a significant depletion of structurally disruptive SNPs among those that actually have been observed in human populations. This is not unexpected since such SNPs are likely to be selected against. On the other hand, no further reduction was found when restricting to the set of disease-associated SNPs. We suspect this to be a consequence of the small fraction of disease-associated SNPs being causal, the rest of them being evolutionary neutral but linked to the causal variation.

At present, the main limitation of the RNAsnp software is its restriction to substitution. Future extensions will include the predictions of structural effects of indels. Although conceptually simple, this requires a substantial extension of tables for the *P*-value computations. In addition, we are planning to combine RNAsnp with comparative structure prediction [Hofacker et al., 2002; Knudsen and Hein, 2003; Seemann et al., 2008] to identify structurally disruptive SNPs in regions in which secondary structure is under stabilizing selection.

## Supplementary material

Disclaimer: Supplementary materials have been peer-reviewed but not copyedited.
