# RNAsnp: Efficient Detection of Local RNA Secondary Structure Changes Induced by SNPs

**DOI:** 10.1002/humu.22323

**Published:** 2013-03-15

**Authors:** Radhakrishnan Sabarinathan, Hakim Tafer, Stefan E Seemann, Ivo L Hofacker, Peter F Stadler, Jan Gorodkin

The original article to which this Erratum refers was published in **Human Mutation** 34(4):546–556 (DOI 10.1002/humu.22273).

In the article cited above, the symbols for *M_ik_* and 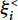 were inadvertently changed during the production process. The corrected text appears below.

The Publisher sincerely regrets this error.

At face value, this minimization is rather expensive because for each index pair *u,v*, the values of π[*u,v*] need to be determined. The naïve evaluation of Eq. (7) can be replaced by a recursive scheme (Supp. Fig. S1). Consider sequence positions *k* <*i* < *l* and denote by *M_ik_* and *N_il_*, the probabilities that *i* has a pairing partner in the interval [*k,i* − 1] and the interval [*i* + 1,*l*], respectively. Clearly, these auxiliary variables satisfy *M_ik_* = *M*_*i(k* − 1)_ + *P_ki_* and *N_il_* = *N*_*i(l* + 1)_ + *P_il_*. Obviously, for all *k* < *i* < *l*, we have 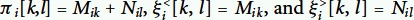. Precomputing *M* and *N* thus allows the evaluation of distances and correlation coefficients in linear time for each sequence interval.

