# Supplementary material for: RNAsnp: Efficient Detection of Local RNA Secondary Structure Changes Induced by SNPs
Source: Hum Mutat. 2013 Jan 11;34(4):546–56. doi: 10.1002/humu.22273 (PMC3708107; doi:10.1002/humu.22273)
Supplement: Supplementary file 1 [file humu0034-0546-SD1.pdf]

Supplementary Materials

RNAsnp: Efficient detection of local RNA secondary structure changes induced by SNPs

Radhakrishnan Sabarinathan, Hakim Tafer, Stefan E. Seemann,
Ivo L. Hofacker, Peter F. Stadler and Jan Gorodkin

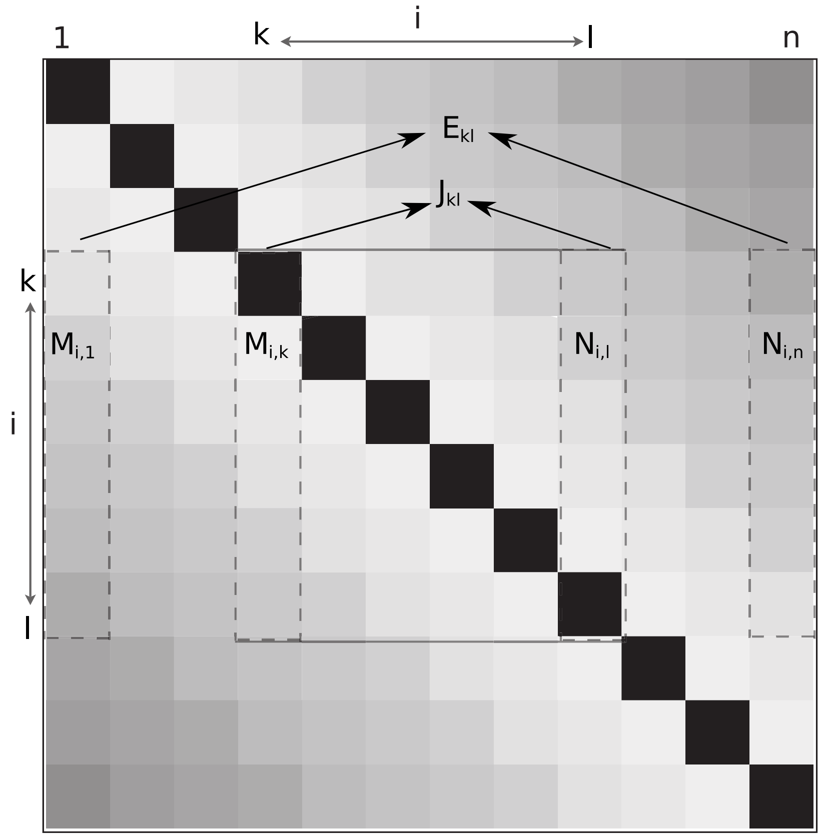

Figure S1 - The graphical representation illustrates the base pairing probability matrix of a sequence. Each cell [i,j] contains the base pairing probability of i with j, i.e. P_{ij}. We employed a recursive method to add all the probabilities along the row, which helps for the faster computation of position-wise pairing probabilities (π). Consider $[k, l]$ is a sequence interval $k < i < l$, and denotes $M_{i,k}$ and $N_{i,l}$ the probabilities that i has a pairing partner in the interval $[k, i-1]$ and the interval $[i+1, l]$, resp. These auxiliary variables satisfy $M_{i,k} = M_{i,k-1} + P_{ki}$ and $N_{i,l} = N_{i,l+1} + P_{il}$. Obviously, for all $k < i < l$ we have $\pi_i[k, l] = M_{i,k} + N_{i,l}$. Also, we can directly compute the expected number of base pairs inside and outside the substructure, J_{kl} and E_{kl}, resp.

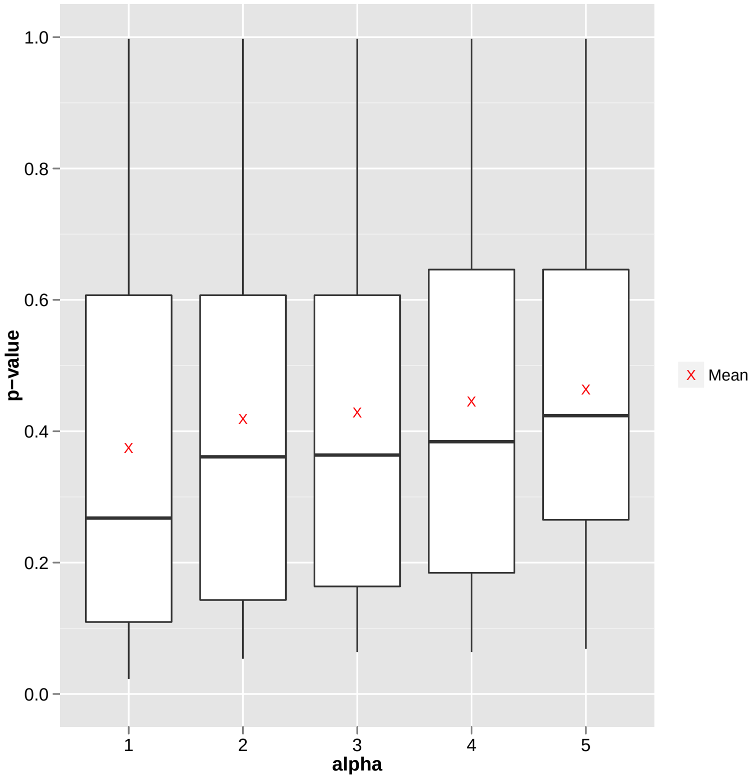

Figure S2 - The data set of 30 SNPs with reported structural effects was used to test the effect of different α values ranging from 1 to 5 in steps of 1. The α parameter determines the ratio between the expected number of base pairs inside (J_{kl}) a local interval $[k, l]$, compared to the expected numbers of base pairs ($E_{k,l}$) cross the boundaries of the interval. The box plot shows the distribution of p-values for the 30 known SNPs changes for the different α values. As expected, the increase in mean p-value correlate with increase in the α value, because the higher the alpha value the greater the increase in expected number of base pairs inside the $[k, l]$ compare to the outside. This eventually results in the selection of larger interval $[k, l]$ for comparison and thus the measure of local structural effect become less significance. Thus, the α=1 is chosen as default.

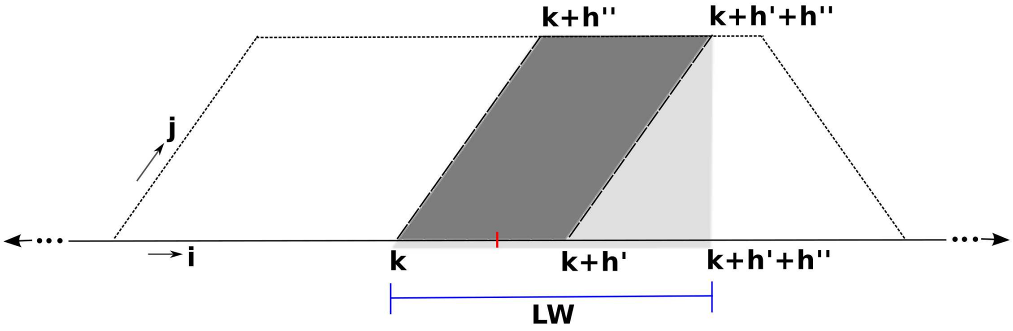

Figure S3 - Schematic representation of `RNAplfold` base pairing probability matrix calculated for a window of 400 nts centering the SNP position. The region highlighted in dark gray is used for the initial screen to find the position k that maximizes $d^2_{(k)}(P, P^*)$. The local window (LW) ranges from k to $k + h' + h''$, where $h' = 20$ and $h'' = 120$. The LW contains the interval $[u, v]$ from which the optimized distance measure ($d^\sharp$) is obtained.

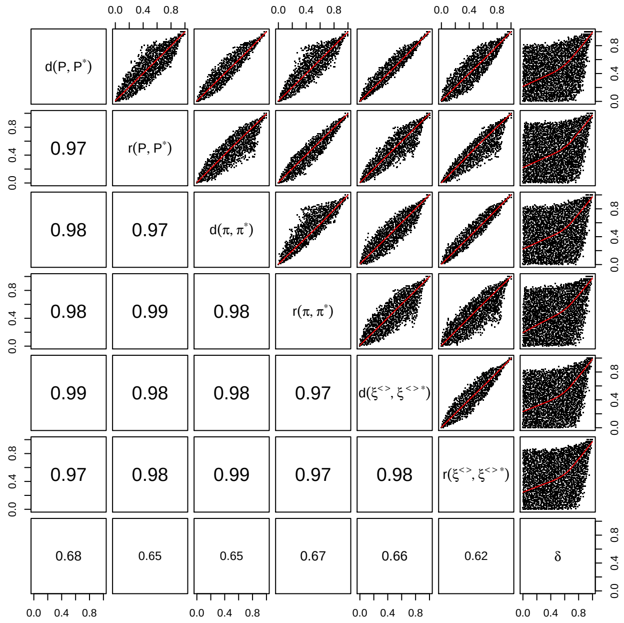

Figure S4 - The correlation between global(dis)similarity measures was computed using the data set of 7000 random sequences of length 400 and considered SNPs at 200 position. The measures distance(d) and correlation coefficient(r) computed on various base pairing probabilities (P - full base pairing probabilities, π - position-wise pairing probabilities, $\xi^{<>}$ - position-wise, distinguished up- and down-stream paring probabilities), correlate with each other. However, these measures does not correlate well with the Euclidean distance (δ) computed between the distribution of wild-type and mutant structures.

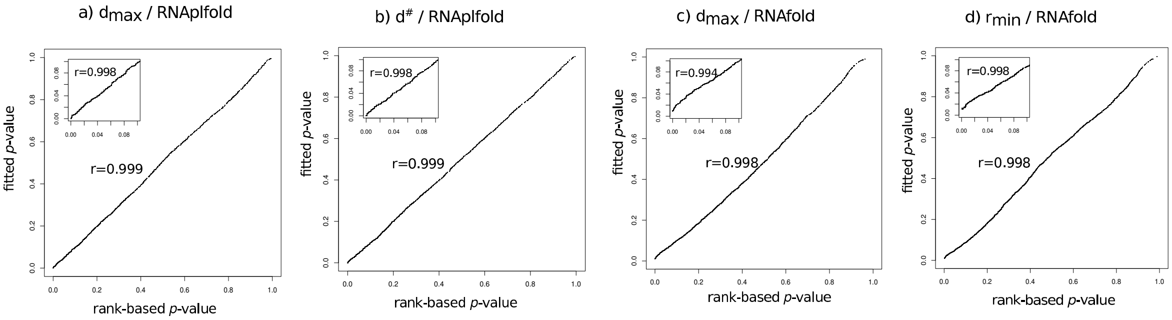

Figure S5 - Comparison of rank-based p-values and fitted p-values for a set of 5000 random numbers. The p-values are calculated from the background distribution of length 400nts, G+C content between 50 and 60% and the SNP position at 200 position. In all four cases, the comparison of rank-based p-value versus the fitted p-value shows high correlation (r>0.9). The inset figure shows the comparison of p-values which are less than 0.1.

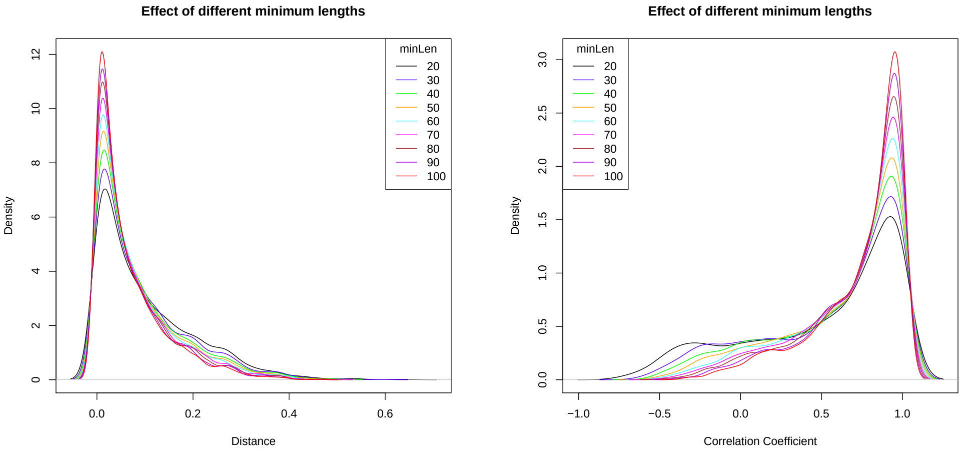

Figure S6 - The effect of minimum length on d_{max}/`RNAfold` and r_{min}/`RNAfold` was tested with different cut-off values. In both cases, the length cut-off 50 shows the unimodal distribution.

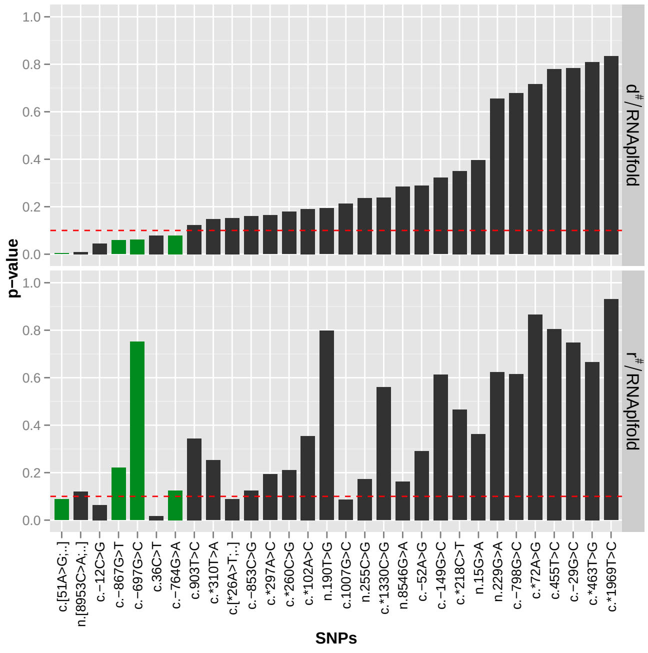

Figure S7 - The significance of structural effects as predicted by $d^{\sharp}$/`RNAplfold` and $r^{\sharp}$/`RNAplfold` for the 30 known SNPs. The p-values are shown as bars and the red dashed line represents the selected threshold value 0.1. The four experimentally validated examples are indicated in green. The SNPs were described according to HGVS nomenclature.

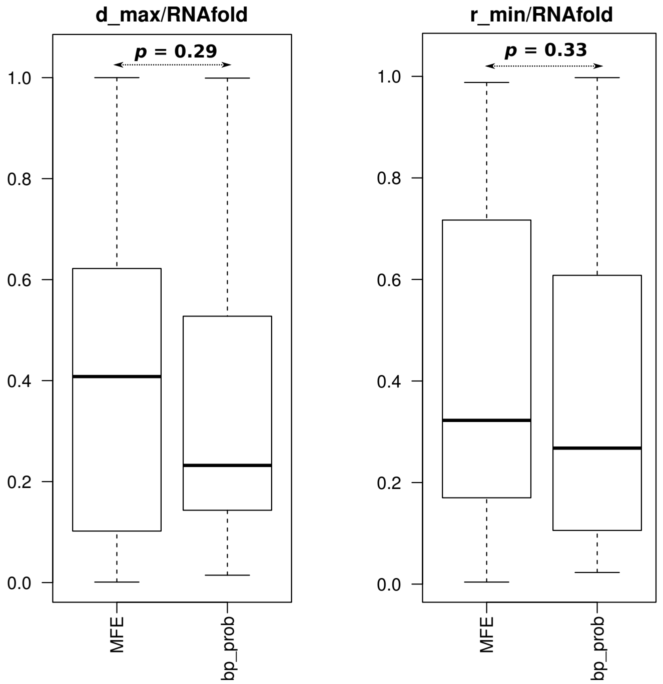

Figure S8 - The data set of 30 SNPs with reported structural effects was used to compare the local measures d_{max} and r_{min} based on base pairing probability of structural ensemble and the base pairs of minimum free energy (MFE) structure, which was calculated using `RNAfold`. The box plot shows the distribution of p-values for the 30 known SNPs obtained for the two measures. The p-values from the local measures based on the structural ensemble are in general smaller than the p-values derived from the MFE structure. The comparison of two p-value distributions using Wilcoxon rank sum test, however, shows no significant difference (P>0.2) for either of the d_{max} or r_{min} measures. This may be explained by the fact that the available data set SNPs are small.

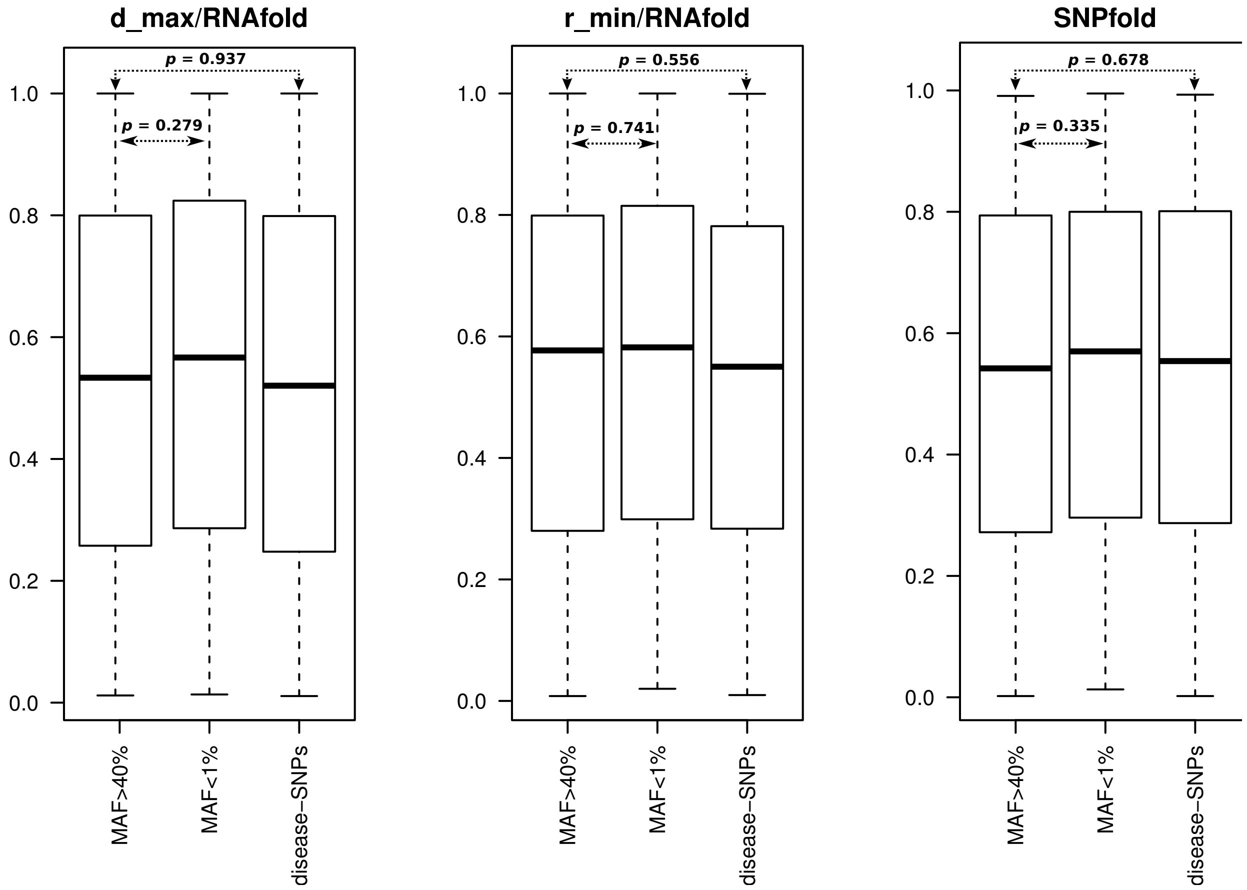

Figure S9 - The box plots show the distribution of p-values calculated for three different data sets that are equal in number of SNPs (n=501) obtained from a) HapMap database with minor allele frequency greater than 40%, b) dbSNP (build 135) with minor allele frequency less than 1% and c) disease-associated SNPs from Human Gene mutation Database (HDMD). The structural effect of these SNPs were predicted with `RNAsnp` (Mode 1) and `SNPfold` [Halvorsen et al., 2010]. For each structural (dis)similarity measure, the difference between the p-value distribution of the three data sets were compared using Wilcoxon rank sum test. It shows that P-value of the wilcoxon rank sum test are higher than the significant level of 0.01 and suggests that there is no significant difference between the p-value distributions of three different SNP data sets. The same scenario was observed in the case of global measure, `SNPfold`.

Table S1 - An overview of 30 SNPs with reported structural effect on RNA secondary structure. The SNPs were described according to HGVS nomenclature.

Disease/Phenotype	Gene	Refseq	SNP	Validation	Reference
Alteration of RNA replication in HCV	NS5B	AJ238799.1	n.[8953C>A;8955T>G]	experimental	[1]
Tumor formation	p53	NM_001126114.2	c.[51A>G;54A>C;57T>C]	experimental	[2]
HIV-1 resistance against RNAi	Nef	K02013.1	n.8546G>A	experimental	[3]
Alteration of alanyl tRNA synthetase expression in human	AARS	D32050.1	c.903T>C	experimental	[4]
Cowden Syndrome	PTEN	NM_000314.4	c.-867G>T	predicted	[5]
			c.-853C>G	predicted	
			c.-798G>C	predicted	
			c.-764G>A	predicted	
Occult-hepatitis B virus infection	-	EU155893.1	n.190T>G	predicted	[6]
Psychiatric disorders	TPH2	NM_173353.3	c.-52A>G	predicted	[7]
Diurnal preference	PER2	NM_022817.2	c.-12C>G	predicted	[8]
Nasopharyngeal Carcinoma Risk	TLR4	NM_138554.4	c.1007G>C	predicted	[9]
Alteration in localization of rat MT1 mRNA	MT1	NM_138826.4	c.[*26A>T;*27G>C; 28G>C;*29T>A;*30G>C]	predicted	[10]
Cone Dystrophy	PDE6H	NM_006205.2	c.-29G>C	predicted	[11]
Congenital heart disease	GATA4	NM_002052.3	c.*260C>G	predicted	[12]
			c.*218C>T	predicted	
Antipsychotic induced weight gain	HTR2C	NM_000868.2	c.-697G>C	predicted	[13]
Muscular dystrophies	SGCG	U34976.1	c.*102A>C	predicted	[14]
Pain sensitivity	COMT	NM_007310.2	c.36C>T	predicted	[15]
Alteration of plasma zymogen TAFI concentration	CPB2	NM_001872.3	c.*310T>A	predicted	[16]
			c.*72A>G	predicted	
Anauxetic dysplasia	RMRP	NR_003051.3	n.255C>G	predicted	[17]
			n.15G>A	predicted	
Mental retardation	CDK5R1	NM_003885.2	c.*1330C>G	predicted	[18]
Alteration of RNA translation in HCV-1b	IRES	EU857431.1	n.229G>A	predicted	[19]
Hyperferritinaemia cataract syndrome	FTL	NM_000146.3	c.-149G>C	predicted	[20]
Resistance to Hirschsprung disease	RET	NM_020975.4	c.*1969T>C	predicted	[21]
Ischemic Cardiomyopathy	ADORA1	NM_000674.2	c.*297A>C	predicted	[22]
Neuropsychiatric disorders	SLC6A4	NM_001045.4	c.*463T>G	predicted	[23]
Cutaneous melanoma	BMP4	NM_001202.3	c.455T>C	predicted	[24]

Results of `Rchange` analysis

In contrast to the RNA mutation analysis programs (like `RNAsnp`) based on base pairing probability, the program Rchange [Kiryu and Asai , 2012] was recently developed to predict the structural effect based on the changes in the energy of RNA secondary structures in response to the single or double mutations. This program was tested on our data set of four known SNPs whose structural effect has been experimentally verified. Since, the program can handle either single or double mutants, only three out of the four known SNPs were tested successfully. `Rchange` computed the changes in thermodynamic entropy (S), mean energy (U) and ensemble free energy (F) between the wild-type and mutant RNA secondary structures. In order to compute the significance value, the RNA sequence of each SNP was shuffled ten times and subjected each of them with `Rchange` to compute the energy difference for the random mutations. Using this result as background distribution, the p-value is estimated using a non-parametric approach for the results of known SNPs (Table S2). Table S2 shows that the `RNAsnp` predicted only one SNP (n.8546G>A) have high structural effect based on the dS/S and $dF/|F|$ measures.

Table S2 - The results of `Rchange` for the three out of four SNPs with reported structural effect. The measures dS/S, $dU/|U|$ and $dF/|F|$ represents, respectively, the difference in the thermodynamic entropy of RNA secondary structures, mean energy and ensemble free energy between the wild-type and mutant RNAs. The p-value with less than 0.1 significance level are highlighted with bold text.

				Rchange			RNAsnp	
				dS/S	$dU/\|U\|$	$dF/\|F\|$	d_{max}/RNAfold	r_{min}/RNAfold
Ref	Gene	Accession	SNP	(p-value)	(p-value)	(p-value)	(p-value)	(p-value)
1	NS5B	AJ238799.1	n.[8953C>A;8955T>G]	0.911	0.802	0.729	**0.074**	0.121
2	AARS	D32050.1	c.903T>C	0.327	0.317	0.303	**0.072**	**0.093**
3	Nef	K02013.1	n.8546G>A	**0.050**	0.590	**0.089**	**0.094**	**0.083**

Table S3 - List of disease associated SNPs from HGMD that are predicted to have significant local structural effect (p-value < 0.1) by d_{max}/`RNAfold` or r_{min}/`RNAfold` of `RNAsnp` (Mode 1). The SNPs were described according to HGVS nomenclature.

Disease/phenotype	Gene	HGMD Accession	Genbank Accession	SNP	p-value: d_{max}/`RNAfold`	p-value: r_{min}/`RNAfold`
Pseudohypoaldosteronism	NR3C2	CR030126	NM_000901.4	c.-2C>G	**0.017**	**0.022**
Hypertension	EDN2	CR994679	NM_001956.3	c.*390G>A	**0.036**	**0.021**
Obesity	CNR1	CR073542	NM_033181.3	c.*2394A>G	**0.032**	**0.036**
Myocardial infarction	GP1BA	CR022116	NM_000173.5	c.-5T>C	**0.040**	**0.037**
Colorectal cancer	INSR	CR082021	NM_001079817.1	c.*104A>G	**0.042**	**0.030**
Graves' disease	FCRL3	CR067134	NM_052939.3	c.-11G>C	**0.011**	**0.042**
Increased triglyceride levels	ABCA1	CR025352	NM_005502.3	c.-279C>G	**0.044**	**0.022**
Insulin resistance hypertension	RETN	CR032443	NM_020415.3	c.*62G>A	**0.045**	**0.043**
Cartilage-Hair hypoplasia	RMRP	CR063417	NR_003051.3	n.215A>G	**0.048**	**0.027**
Hypercholesterolaemia	LDLR	CR971948	NM_000527.4	c.-14C>A	**0.025**	**0.048**
Glaucoma	CYP1B1	CR032431	NM_000104.3	c.-286C>T	**0.063**	**0.036**
Reduced transcriptional activity	NR3C1	CR016150	NM_001024094.1	c.-219C>A	**0.044**	**0.063**
HDL cholesterol levels	LIPG	CR032437	NM_006033.2	c.*482A>G	**0.051**	**0.065**
Factor VII deficiency	F7	CR090334	NM_019616.2	c.-44T>C	**0.066**	**0.042**
HaemophiliaA	F8	CR070421	NM_000132.3	c.-112G>A	**0.074**	**0.010**
Cartilage-Hair hypoplasia	RMRP	CR064472	NR_003051.3	n.10T>C	**0.076**	**0.024**
VonHippel-Lindau syndrome	VHL	CR011856	NM_000551.3	c.*7C>G	**0.076**	**0.065**
Obesity	SLC6A14	CR035766	NM_007231.3	c.*178C>G	**0.078**	**0.062**
Spasticparaplegia31	REEP1	CR082030	NM_022912.2	c.*14C>T	**0.033**	**0.081**
Hyperferritinaemia-cataract syndrome	FTL	CR061334	NM_000146.3	c.-178T>G	**0.052**	**0.097**
Severe iron overload	ALAS2	CR090059	NM_001037968.3	c.-69C>T	**0.078**	0.390
Systemic lupus erythematosus	CRP	CR040151	NM_000567.2	c.*1082G>A	**0.048**	0.316
Migraine	EDNRA	CR011854	NM_001957.3	c.-67G>A	**0.053**	0.106
Hyperferritinaemia-cataract syndrome	FTL	HR030029	NM_000146.3	c.-171C>G	**0.100**	0.354
Cholesterol level	GHRL	CR065638	NR_024132.1	n.316G>C	**0.056**	0.110
Colorectal cancer	MLH1	CR033148	NM_000249.3	c.-28A>T	**0.097**	0.140
Panencephalitis	MX1	CR040301	NM_002462.3	c.-434G>T	**0.058**	0.258
Lipoprotein/Triglyceride levels	PCK1	CR054265	NM_002591.3	c.*431T>C	**0.084**	0.103
Cowden disease	PTEN	CR032094	NM_000314.4	c.-930G>A	**0.019**	0.109
Diabetes	PTEN	CR033149	NM_000314.4	c.-8C>G	**0.098**	0.266
Chronic obstructive pulmonary disease	SERPINA1	CR061339	NM_001127701.1	c.-458C>T	**0.079**	0.129
Haemochromatosis	SLC40A1	CR057017	NM_014585.5	c.-187A>G	**0.045**	0.282

Table S3 - continued

Disease/phenotype	Gene	HGMD Accession	Genbank Accession	SNP	*p*-value d_{max}/RNAfold	*p*-value r_{min}/RNAfold
Aplastic anaemia	TERC	CR057475	NR_001566.1	n.117A>C	**0.035**	0.223
Aplastic anaemia	TERC	CR080776	NR_001566.1	n.2G>C	**0.082**	0.198
Chondrocalcinosis	ANKH	CR057902	NM_054027.4	c.-4G>A	0.131	**0.029**
Factor XI deficiency	F11	CR064469	NM_000128.3	c.-54G>A	0.124	**0.069**
Factor VII deficiency	F7	CR002894	NM_019616.2	c.-30A>C	0.150	**0.076**
IPEX syndrome	FOXP3	CR063404	NM_001114377.1	c.-7G>T	0.116	**0.067**
Decreased expression	GCH1	CR075245	NM_000161.2	c.*243C>T	0.148	**0.065**
Frontotemporal dementia?	GRN	CR072310	NM_002087.2	c.-72G>T	0.178	**0.054**
Hypercholesterolaemia	LDLR	CR042574	NM_000527.4	c.-153C>T	0.116	**0.059**
Hypercholesterolaemia	LDLR	CR951555	NM_000527.4	c.-138T>C	0.170	**0.091**
Cellular response to cadmium	MT2A	CR066330	NM_005953.3	c.-77A>G	0.126	**0.053**
Reduced expression	NEIL2	CR085800	NM_145043.2	c.-586C>G	0.126	**0.091**
Schizophrenia	NOS1	CR025919	NM_000620.4	c.*276C>T	0.181	**0.059**
Decr.serum leptin levels in lean indiv.	POMC	CR035490	NM_000939.2	c.*63C>T	0.227	**0.092**
Hirschsprung disease	RET	CR951557	NM_020975.4	c.-27C>G	0.173	**0.095**
Cartilage-Hair hypoplasia	RMRP	CR012677	NR_003051.3	n.263G>T	0.130	**0.051**
	RMRP	CR021393	NR_003051.3	n.183G>C	0.135	**0.085**
	RMRP	CR021394	NR_003051.3	n.212C>G	0.182	**0.067**
	RMRP	CR054268	NR_003051.3	n.183G>T	0.136	**0.085**
	RMRP	CR054277	NR_003051.3	n.214C>G	0.108	**0.039**
Pancreatitis	SPINK1	CR001469	NM_003122.3	c.-53C>T	0.121	**0.020**
Nasopharyngeal cancer	TLR4	CR068105	NR_024168.1	n.3938G>C	0.210	**0.083**

Table S4 - List of disease associated SNPs from GWASdb that are predicted to have significant local structural effect by d_{max}/`RNAplfold` ($p < 0.4$) and d_{max}/`RNAfold` ($p < 0.1$) of `RNAsnp` (with mode 3). The SNPs were described according to HGVS nomenclature.

Disease/phenotype	Ensembl id	UTR	dbSNP	p-value d_{max}/`RNAfold`	p-value r_{min}/`RNAfold`
Suicide attempts in bipolar disorder	ENST00000373055	3	rs7822:T>C	0.0290	0.0199
Lapatinib-induced hepatotoxicity	ENST00000360403	5	rs489676:C>G	0.0391	0.0925
Alzheimer‘s disease (late onset)	ENST00000368485	3	rs7514452:C>T	0.1847	0.0984
Multiple complex diseases	ENST00000368476	3	rs11264221:C>T	0.1611	0.0356
Ischemic stroke;Stroke	ENST00000329117	3	rs11360:A>G	0.0594	0.0514
Systemic lupus erythematosus	ENST00000255030	3	rs1205:G>A	0.2241	0.0477
Suicide attempts in bipolar disorder	ENST00000333360	3	rs5357:T>C	0.0896	0.0296
Alcohol dependence	ENST00000319387	3	rs4233175:A>G	0.3642	0.0883
Sudden cardiac arrest	ENST00000260585	3	rs3820937:G>C	0.2935	0.0826
GWAS of height-adjusted highest forced expiratory volume in a British population	ENST00000379066	3	rs1056021:T>C	0.0021	0.0074
Urinary metabolites	ENST00000426016	3	rs6704656:T>A	0.2238	0.0903
Amyotrophic Lateral Sclerosis (ALS)	ENST00000306448	5	rs896210:C>T	0.0394	0.0739
Multiple complex diseases	ENST00000306503	3	rs17823065:T>C	0.0364	0.0249
Parkinson‘s disease; Multiple complex diseases	ENST00000254630	3	rs11395:T>C	0.3539	0.0370
Urinary metabolites	ENST00000259213	3	rs4849142:C>T	0.0335	0.0690
Parkinson‘s disease	ENST00000338983	3	rs8446:T>C	0.0482	0.0939
Multiple complex diseases	ENST00000443029	5	rs2290536:T>G	0.3384	0.0222
Urinary metabolites	ENST00000357632	3	rs17765088:C>G	0.0043	0.0108
Lung adenocarcinoma	ENST00000433104	3	rs3172494:C>A	0.0015	0.0166
Serum calcium	ENST00000344337	3	rs17201246:G>T	0.3573	0.0712
Multiple continuous traits in DGI samples	ENST00000305097	3	rs16864613:C>G	0.1268	0.0405
Alzheimer‘s disease (late onset)	ENST00000337774	3	rs3821801:A>G	0.0123	0.0753
Serum uric acid;Serum urate	ENST00000326756	3	rs3217:G>A	0.0439	0.0363
GWAS of systolic blood pressure in a British population	ENST00000344157	3	rs2293595:A>G	0.1178	0.0504
Serum uric acid	ENST00000237596	3	rs2728121:C>T	0.0631	0.0182
Parkinson‘s disease	ENST00000394989	3	rs3857053:G>A	0.1326	0.0566
Alcohol dependence	ENST00000515683	3	rs2298753:A>G	0.0012	0.0072
Multiple complex diseases	ENST00000285311	3	rs17509643:C>G	0.1033	0.0770
potassium response to spironolactone	ENST00000355292	5	rs2070951:C>G	0.0466	0.0167
Alzheimer‘s disease (late onset)	ENST00000061240	3	rs2279723:C>A	0.0611	0.0564
Alzheimer‘s disease (late onset)	ENST00000284274	3	rs25952:A>C	0.1341	0.0659
Thrombosis	ENST00000356834	3	rs1298:C>T	0.0129	0.0907

Table S4 - continued

	Ensembl			*p*-value	
Disease/phenotype	id	UTR	dbSNP	d_{max}/RNAfold	r_{min}/RNAfold
Common traits (Other)	ENST00000380956	3	rs1050975:G>A	0.1541	0.0154
Phospholipid levels (plasma)	ENST00000354666	3	rs4532436:G>C	0.0171	0.0486
Multiple complex diseases	ENST00000383555	3	rs2073149:A>T	0.0943	0.0114
Lung adenocarcinoma;Rheumatoid Arthritis	ENST00000376883	5	rs2535238:G>T	0.0419	0.0468
Rheumatoid arthritis;Lung cancer	ENST00000449742	3	rs2257914:G>T	0.0303	0.0103
Rheumatoid Arthritis	ENST00000375015	3	rs482194:T>C	0.0844	0.0795
Rheumatoid Arthritis	ENST00000395388	5	rs14004:C>A	0.2745	0.0265
Serum metabolites; Multiple complex diseases; Multiple sclerosis	ENST00000395388	3	rs7194:G>A	0.3531	0.0417
HIV-1 control	ENST00000374897	3	rs241454:T>C	0.1504	0.0461
Multiple complex diseases	ENST00000374680	3	rs2744537:T>G	0.1538	0.0268
Multiple complex diseases	ENST00000482399	3	rs461338:A>G	0.1284	0.0420
Prostatic Neoplasms;height	ENST00000311565	5	rs2016520:C>T	0.2705	0.0659
Lipoprotein-associated phospholipase A2 activity and mass	ENST00000544460	3	rs12528857:C>A	0.3672	0.0645
Attention Deficit Disorder with Hyperactivity	ENST00000369838	3	rs1062793:A>G	0.0807	0.0925
Coronary heart disease;	ENST00000367882	3	rs12190287:C>G	0.0345	0.0622
Bone mineral density (spine)	ENST00000367290	3	rs6932603:T>C	0.1815	0.0227
Multiple complex diseases	ENST00000222792	3	rs1420145:C>G	0.0090	0.0138
Aortic root size;Aortic root size	ENST00000360415	3	rs875971:T>C	0.2875	0.0137
Major depressive disorder	ENST00000005178	3	rs11531570:C>T	0.3857	0.0633
Alzheimer‘s disease	ENST00000205402	3	rs4564:G>A	0.1477	0.0272
Glaucoma (primary open-angle)	ENST00000222693	3	rs1052990:T>G	0.0436	0.0439
Inflammatory Bowel Diseases	ENST00000466675	3	rs4721:A>C	0.0507	0.0936
Information processing speed	ENST00000401878	3	rs2007922:G>A	0.0018	0.0169
Multiple complex diseases	ENST00000256255	3	rs3189926:T>G	0.1546	0.0134
Multiple continuous traits in DGI samples	ENST00000342228	3	rs6841:C>G	0.0352	0.0934
Suicide attempts in bipolar disorder	ENST00000289957	5	rs4950:G>A	0.0926	0.0633
Suicide attempts in bipolar disorder	ENST00000521271	3	rs1044730:C>T	0.0089	0.0812
Amyotrophic Lateral Sclerosis (ALS)	ENST00000297848	3	rs2429:G>T	0.1519	0.0903
Parkinson‘s disease	ENST00000314393	3	rs3802266:A>G	0.3048	0.0906
Other erythrocyte phenotypes; Multiple complex diseases	ENST00000381750	3	rs1053872:G>C	0.0308	0.0516
Parkinson‘s disease (age of onset)	ENST00000374193	3	rs10817478:A>G	0.0629	0.0215
Proinsulin levels	ENST00000372155	3	rs306549:G>C	0.0282	0.0752
Achilles tendinopathy	ENST00000371817	3	rs12722:C>T	0.1740	0.0536

Table S4 - continued

Disease/phenotype	Ensembl id	UTR	dbSNP	*p*-value d_{max}/RNAfold	*p*-value r_{min}/RNAfold
Multiple complex diseases	ENST00000446108	3	rs8463:A>G	0.0289	0.0987
Cognitive test performance; Hirschsprung's disease	ENST00000355710	3	rs17028:C>T	0.0768	0.0330
Urinary metabolites	ENST00000333254	5	rs41386650:A>G	0.1587	0.0961
Type II Diabetes Mellitus	ENST00000396952	3	rs10500609:A>G	0.0848	0.0705
Rheumatoid Arthritis, cyclic citrullinated peptide (CCP) positive	ENST00000318950	3	rs360136:C>A	0.0226	0.0651
Multiple complex diseases	ENST00000361905	3	rs16911839:C>G	0.0188	0.0363
Amyloid A Levels	ENST00000396253	3	rs12416821:T>C	0.0472	0.0243
Type II Diabetes Mellitus	ENST00000327470	3	rs10500938:C>T	0.3011	0.0968
Urinary metabolites	ENST00000321505	3	rs7123662:A>T	0.0837	0.0352
GWAS of log10 serum total immunoglobulin E concentration in a British population	ENST00000353172	3	rs3824865:A>G	0.0716	0.0923
Multiple complex diseases	ENST00000279441	3	rs470171:C>G	0.0841	0.0443
Cardiovascular disease risk factors	ENST00000530849	3	rs1047964:G>C	0.1902	0.0221
Multiple complex diseases	ENST00000357529	3	rs9783460:C>A	0.3374	0.0609
Alpha-2-Macroglobulin	ENST00000318602	5	rs226380:T>G	0.0111	0.0445
Multiple continuous traits in DGI samples	ENST00000486433	3	rs2638315:C>G	0.0073	0.0597
Multiple continuous traits in DGI samples	ENST00000378485	3	rs17251627:A>G	0.2998	0.0355
Insulin resistance/response	ENST00000360185	3	rs7314498:G>A	0.0363	0.0663
Type 1 diabetes; Multiple complex diseases	ENST00000550722	3	rs3519:C>T	0.2340	0.0653
Suicide attempts in bipolar disorder	ENST00000382298	5	rs17078720:A>G	0.3550	0.0404
Parkinson's disease	ENST00000554271	3	rs7560:T>G	0.1258	0.0516
Multiple complex diseases	ENST00000335725	3	rs9488:C>T	0.0698	0.0283
Breast Neoplasms	ENST00000396402	3	rs4646:T>G	0.2089	0.0501
Smoking behavior;Lung cancer; Chronic obstructive pulmonary disease;Lung adenocarcinoma	ENST00000258886	3	rs1062980:T>C	0.0270	0.0121
Smoking behavior;	ENST00000044462	5	rs3813570:T>C	0.0043	0.0542
Lung adenocarcinoma	ENST00000261751	3	rs1948:T>C	0.0888	0.0615
Longevity	ENST00000284382	3	rs12914235:A>G	0.2592	0.0202
Insulin-like growth factors	ENST00000262302	3	rs1065656:C>G	0.0116	0.0687
Insulin resistance/response	ENST00000318282	3	rs30126:C>T	0.1501	0.0382
Multiple complex diseases	ENST00000540146	3	rs1054028:T>C	0.0105	0.0231
Hemoglobin A, Glycosylated	ENST00000324015	3	rs1057355:G>T	0.1312	0.0889
Allergic rhinitis	ENST00000322957	3	rs3192453:G>C	0.2871	0.0622
Myopia (pathological)	ENST00000306329	3	rs3744975:C>T	0.0033	0.0194

Table S4 - continued

Disease/phenotype	Ensembl id	UTR	dbSNP	*p*-value d_{max}/`RNAfold`	*p*-value r_{min}/`RNAfold`
Bladder cancer	ENST00000436407	5	rs10432193:T>C	0.0618	0.0533
Multiple continuous traits in DGI samples	ENST00000334889	3	rs9947104:T>C	0.0199	0.0908
Gallstone disease	ENST00000251047	3	rs1043334:A>C	0.2259	0.0403
Alcohol dependence	ENST00000302850	3	rs1864193:G>T	0.1657	0.0978
Suicide attempts in bipolar disorder	ENST00000222249	3	rs1057261:A>G	0.1008	0.0751
Urinary Tract Infections; Vesico-Ureteral Reflux	ENST00000243578	3	rs1800468:G>A	0.1071	0.0814
Alcohol dependence;	ENST00000300843	3	rs344797:T>G	0.0037	0.0745
Multiple continuous traits in DGI samples	ENST00000262919	3	rs432647:T>C	0.1697	0.0284
Suicide attempts in bipolar disorder	ENST00000246006	3	rs7492:T>C	0.0380	0.0311
Plasma levels of Protein C	ENST00000246186	3	rs6060341:A>G	0.3550	0.0963
Parkinson‘s disease;	ENST00000244061	3	rs6125829:G>T	0.0446	0.0674
GWAS of bipolar disorder in the Japanese population	ENST00000284987	3	rs229070:C>G	0.3589	0.0856

References

[1] You S, Stump DD, Branch AD, Rice CM. 2004. A *cis*-acting replication element in the sequence encoding the NS5B RNA-dependent RNA polymerase is required for hepatitis C virus RNA replication. J Virol 78:1352-1366.

[2] Grover R, Sharathchandra A, Ponnuswamy A, Khan D, Das S. 2011. Effect of mutations on the p53 IRES RNA structure: Implications for de-regulation of the synthesis of p53 isoforms. RNA Biol 8:132-142.

[3] Westerhout EM, Ooms M, Vink M, Das AT, Berkhout B. 2005. HIV-1 can escape from RNA interference by evolving an alternative structure in its RNA genome. Nucleic Acids Res 33:796-804.

[4] Shen LX, Basilion JP, Stanton VP. 1999. Single-nucleotide polymorphisms can cause different structural folds of mRNA. Proc Natl Acad Sci U S A, 96:7871-7876.

[5] Teresi RE, Zbuk KM, Pezzolesi MG, Waite KA, Eng C. 2007. Cowden syndrome-affected patients with PTEN promoter mutations demonstrate abnormal protein translation. Am J Hum Genet, 81:756-767.

[6] van Hemert FJ, Zaaijer HL, Berkhout B, Lukashov VV. 2008. Occult hepatitis B infection: an evolutionary scenario. Virology J 5:146.

[7] Chen GL, Vallender EJ, Miller GM. 2008. Functional characterization of the human TPH2 5' regulatory region: untranslated region and polymorphisms modulate gene expression in vitro. Hum Genet 122:645-657.

[8] Carpen JD, Archer SN, Skene DJ, Smits M, von Schantz M. 2005. A single-nucleotide polymorphism in the 5'-untranslated region of the hPER2 gene is associated with diurnal preference. J Sleep Res 14:293-297.

[9] Song C, Chen LZ, Zhang RH, Yu XJ, Zeng YX. 2006. Functional variant in the 3'-untranslated region of Toll-like receptor 4 is associated with nasopharyngeal carcinoma risk. Cancer Biol Ther, 5:1285-1291.

[10] Nury D, Chabanon H, Levadoux-Martin M, Hesketh J.. 2005. An eleven nucleotide section of the 3'-untranslated region is required for perinuclear localization of rat metallothionein-1 mRNA. Biochem J 387:419-428.

[11] Piri N, Gao YQ, Danciger M, Mendoza E, Fishman GA, Farber DB. 2005. A substitution of G to C in the cone cGMP-phosphodiesterase gamma subunit gene found in a distinctive form of cone dystrophy. Ophthalmology, 112:159-166.

[12] Reamon-Buettner SM, Cho SH, Borlak J. 2007. Mutations in the 3'-untranslated region of GATA4 as molecular hotspots for congenital heart disease (CHD). BMC Med Genet 8:38.

[13] Hill MJ, Reynolds GP. 2011. Functional consequences of two HTR2C polymorphisms associated with antipsychotic-induced weight gain. Pharmacogenomics, 12:727-734.

[14] Siala O, Salem IH, Tlili A, Ammar I, Belguith H, Fakhfakh F. 2010. Novel sequence variations in LAMA2 and SGCG genes modulating cis-acting regulatory elements and RNA secondary structure. Genet Mol Bio 33:190-197.

[15] Tsao D, Shabalina SA, Gauthier J, Dokholyan NV, Diatchenko L. 2011. Disruptive mRNA folding increases translational efficiency of catechol-O-methyltransferase variant. Nucleic Acids Res 39:6201-6212.

[16] Boffa MB, Maret D, Hamill JD, Bastajian N, Crainich P, Jenny NS, Tang Z, Macy EM, Tracy RP, Franco RF, Nesheim ME, Koschinsky ML. 2008. Effect of single nucleotide polymorphisms on expression of the gene encoding thrombin-activatable fibrinolysis inhibitor: a functional analysis. Blood, 111:183-189.

[17] Thiel CT, Horn D, Zabel B, Ekici AB, Salinas K, Gebhart E, Rschendorf F, Sticht H, Spranger J, Mller D, Zweier C, Schmitt ME, Reis A, Rauch A. 2005. Severely incapacitating mutations in patients with extreme short stature identify RNA-processing endoribonuclease RMRP as an essential cell growth regulator. Am J Hum Genet 77:795-806.

[18] Venturin M, Moncini S, Villa V, Russo S, Bonati MT, Larizza L, Riva P. 2006. Mutations and novel polymorphisms in coding regions and UTRs of CDK5R1 and OMG genes in patients with non-syndromic mental retardation. Neurogenetics, 7:59-66.

[19] Tang S, Collier AJ, Elliott RM. 1999. Alterations to both the primary and predicted secondary structure of stem-loop IIIc of the hepatitis C virus 1b 5' untranslated region (5'UTR) lead to mutants severely defective in translation which cannot be complemented in trans by the wild-type 5'UTR sequence. J Virol 73:2359-2364.

[20] Camaschella C, Zecchina G, Lockitch G, Roetto A, Campanella A, Arosio P, Levi S. 2000. A new mutation (G51C) in the iron-responsive element (IRE) of L-ferritin associated with hyperferritinaemia-cataract syndrome decreases the binding affinity of the mutated IRE for iron-regulatory proteins. Br J Haematol 108:480-482.

[21] Griseri P, Lantieri F, Puppo F, Bachetti T, Di Duca M, Ravazzolo R, Ceccherini I. 2007 A common variant located in the 3'UTR of the RET gene is associated with protection from Hirschsprung disease. Hum Mutat 28:168-76.

[22] Tang Z, Diamond MA, Chen JM, Holly TA, Bonow RO, Dasgupta A, Hyslop T, Purzycki A, Wagner J, McNamara DM, Kukulski T, Wos S, Velazquez EJ, Ardlie K, Feldman AM. 2007. Polymorphisms in adenosine receptor genes are associated with infarct size in patients with ischemic cardiomyopathy. Clin Pharmacol Ther 82:435-440.

[23] Vallender EJ, Priddy CM, Hakim S, Yang H, Chen GL, Miller GM. 2008. Functional variation in the 3' untranslated region of the serotonin transporter in human and rhesus macaque. Genes Brain Behav 7:690-697.

[24] Capasso M, Ayala F, Russo R, Avvisati RA, Asci R, Iolascon A. 2009. A predicted functional single-nucleotide polymorphism of bone morphogenetic protein-4 gene affects mrna expression and shows a significant association with cutaneous melanoma in southern italian population. J Cancer Res Clin Oncol 135:1799-1807.
